# Identification, Characterization, Pathogenicity, and Fungicide Sensitivity of Postharvest Fungal Diseases in Culinary Melon from Northern Thailand

**DOI:** 10.3390/jof11070540

**Published:** 2025-07-19

**Authors:** Nakarin Suwannarach, Karnthida Wongsa, Chanokned Senwanna, Wipornpan Nuangmek, Jaturong Kumla

**Affiliations:** 1 Office of Research Administration, Chiang Mai University, Chiang Mai 50200, Thailand; chanokned.swn@gmail.com (C.S.); jaturong_yai@hotmail.com (J.K.); 2 Center of Excellence in Microbial Diversity and Sustainable Utilization, Chiang Mai University, Chiang Mai 50200, Thailand; karnthida.wongsa@gmail.com; 3 Department of Biology, Faculty of Science, Chiang Mai University, Chiang Mai 50200, Thailand; 4 School of Agriculture and Natural Resources, University of Phayao, Phayao 56000, Thailand; wipornpan.nu@up.ac.th

**Keywords:** agricultural crop, fungal disease, fungicide sensitivity, pathogen identification, postharvest disease

## Abstract

Culinary melon (*Cucumis melo* subsp. *agrestis* var. *conomon*) is widely cultivated throughout Thailand and represents an important agricultural crop. During 2023–2024, anthracnose, charcoal rot, and fruit rot caused by fungi were observed on postharvest culinary melon fruits in northern Thailand. This study aimed to isolate and identify fungal pathogens associated with these postharvest diseases in culinary melons, as well as to assess their pathogenicity. Eight fungal strains were isolated and identified through morphological characterization and multi-gene phylogenetic analysis. *Colletotrichum chlorophyti* and *C. siamense* were identified as the causal agents of anthracnose, *Fusarium sulawesiense* caused fruit rot, and *Macrophomina phaseolina* was responsible for charcoal rot. Pathogenicity tests were conducted, and the fungi were successfully re-isolated from the symptomatic lesions. Moreover, sensitivity tests for fungicides revealed that *C. siamense* was completely inhibited by copper oxychloride and copper hydroxide. *Colletotrichum chlorophyti* was inhibited by benalaxyl-M + mancozeb, copper hydroxide, and mancozeb. In the case of *M. phaseolina*, complete inhibition was observed with the use of benalaxyl-M + mancozeb, mancozeb, and propineb. Copper hydroxide successfully inhibited *F. sulawesiense* completely. To our knowledge, this study is the first to report *C. siamense* and *C. chlorophyti* as causes of anthracnose, *F. sulawesiense* as a cause of fruit rot, and *M. phaseolina* as a cause of charcoal rot in postharvest culinary melon fruits in Thailand. It also marks the first global report of *C. siamense*, *M. phaseolina*, and *F. sulawesiense* as causal agents of these respective diseases in culinary melon. Furthermore, the results of the fungicide sensitivity tests provide valuable information for developing effective management strategies to control these postharvest diseases in the future.

## 1. Introduction

In 2023, the global production of cantaloupes and other melons reached approximately 29.5 million tons, with an estimated value of USD 15.2 billion [1]. Asia was the leading producing region, contributing 22.3 million tons, followed by the Americas (3.8 million tons), Europe (1.9 million tons), Africa (1.2 million tons), and Oceania (0.2 million tons). Among individual countries, China was the top producer with 14.4 million tons, followed by India and Tunisia, each producing 1.4 million tons, and Kazakhstan with 1.3 million tons [1]. In Southeast Asia, Indonesia was the largest producer of cantaloupes and other melons, followed by the Philippines and the Lao People’s Democratic Republic [1]. Cantaloupes and other melon varieties are highly susceptible to a broad spectrum of bacterial, fungal, and viral diseases throughout the cultivation cycle, during harvest, and in the postharvest storage stages [2,3,4,5,6,7]. Pathogenic fungi are detrimental disease agents that affect cantaloupes and other melon fruits both preharvest and postharvest, resulting in significant reductions in productivity and quality, leading to customer dissatisfaction and economic losses. Previous investigation indicates that fungi corresponding to the genera *Alternaria* [8], *Diaporthe* [9], *Fusarium* [4,7], *Lasiodiplodia* [10], *Neoscytalidium* [11], *Paramyrothecium* [12], *Penicillium* [13], *Rhizopus* [14], *Sclerotium* [15], and *Stagonosporopsis* [16,17] have been associated with fruit rot in cantaloupes and other melons. Charcoal rot can be a serious postharvest disease in cucurbits, including melons, with *Macrophomina phaseolina* identified as the primary causal agent [18,19,20]. Additionally, anthracnose disease on cantaloupes and other melons, caused by the *Colletotrichum* species, is considered one of the most significant diseases affecting these crops [3,21,22]. These diseases have become important postharvest diseases worldwide. Therefore, losses in cantaloupes and other melons due to these diseases can be reduced through appropriate management strategies, accurate disease diagnosis, and efficient disease detection.

Culinary melon (*Cucumis melo* subsp. *agrestis* var. *conomon*) is one of the commercially significant horticultural plants within the family Cucurbitaceae. It is a popular vegetable crop cultivated in China, India, Japan, Korea, and Southeast Asia [23,24,25]. Culinary melon is recognized for its nutritional values (protein, carbohydrates, dietary fiber, minerals, and vitamins) and medicinal properties (anticancer, antioxidant, and antimutagenic activities) for humans [25,26]. Varied bioactive compounds, including fragrant compounds, polyphenols, and phytosterols, have been isolated from fruits of culinary melon [25,27]. Currently, the production of culinary melons and other types of melons is expanding in Thailand, making them one of the country’s most important agricultural crops. Culinary melon is known as “Taeng-Thai” or “Taeng-Lai”, and its mature fruits are eaten fresh or cooked in sour curry in Thailand. It is grown in the northern Thai Provinces of Chiang Mai, Chiang Rai, Nakhon Sawan, Phayao, Phrae, Phitsanulok, and Sukhothai. Prior to this present study, postharvest fruit rot caused by *Fusarium* species has been reported in cantaloupes and muskmelons in Thailand [5,7,10,28,29]. However, there have been no reports on the fungal agents causing postharvest diseases of culinary melon in Thailand. In this study, postharvest diseases of culinary melon caused by fungi were surveyed in 2023 and 2024 (from February to April and from December to January) in the Chiang Mai, Chiang Rai, and Phayao Provinces, northern Thailand. Based on the number of fruits (100 fruits per pallet box) in each pallet box, the disease incidence varied between 10% and 25%. As a result, a significant portion of the fruit crop could not be sold. Therefore, there is still a need to identify the causative agents of these diseases. The purpose of this study was to isolate, identify, and evaluate the pathogenicity of the fungal causal agents. Morphological characteristics, combined with a multigene phylogenetic analysis, were used to identify the obtained fungi. Additionally, the application of Koch’s postulates verified the pathogenicity. Furthermore, the sensitivity of the isolated fungi to commercial fungicides was also examined.

## 2. Materials and Methods

### 2.1. Sample Collection

During the postharvest storage, culinary melon (*Cucumis melo* subsp. *agrestis* var. *conomon*) fruits harvested at the fully ripe stage from Chiang Mai, Chiang Rai, and Phayao Provinces in northern Thailand exhibited anthracnose, charcoal rot, and fruit rot throughout 2023 and 2024. The storage conditions ranged from 26 to 34 °C with a relative humidity between 60 and 75% for periods of 7 to 14 days. Ten fruits exhibiting typical symptoms were randomly collected, placed in sterile plastic boxes, and transported to the laboratory within 24 h. After the symptomatic fruits arrived at the laboratory, they were examined under a stereo microscope (Nikon H55OS, Tokyo, Japan) and then stored in a plastic container with moist filter paper to promote sporulation.

### 2.2. Fungal Isolation

Fungal causal agents were isolated from the margin of the lesions using the tissue transplant method [30]. This process was carried out on potato dextrose agar (PDA; Conda, Madrid, Spain) supplemented with streptomycin at a final concentration of 0.5 mg/L [7]. The isolated plates were incubated at 25 °C in the dark. After 24 to 48 h, the hyphal tips were transferred to PDA supplemented with 0.5 mg/L streptomycin. Each pure fungal strain was purified and subcultured on PDA. Each strain was preserved by storing on PDA slants at 4 °C for short-term preservation (up to 6 months) and in 20% glycerol at –80 °C for long-term preservation (over 6 months to several years). All fungal strains obtained in this study were maintained at the Sustainable Development of Biological Resources culture collection, Faculty of Science, Chiang Mai University (SDBR-CMU), located in Chiang Mai Province, Thailand.

### 2.3. Fungal Identification

#### 2.3.1. Morphological Study

Morphological characteristics were determined according to the previous methods [19,30,31,32,33]. The characteristic morphology, pigmentation, and odor of fungal colonies were examined on PDA after incubation at 25 °C in darkness for seven days. Micromorphological characteristics were examined under a light microscope (Nikon Eclipse Ni-U, Tokyo, Japan). At least 50 measurements were made for each micromorphological structure (e.g., conidiophore, conidiogenous cell, conidia, and chlamydospore) using the Tarosoft (R) Image Frame Work version 0.9.7 program.

#### 2.3.2. DNA Extraction, PCR Amplification, and Sequencing

The Fungal DNA Extraction Kit (FAVORGEN, Ping-Tung, Taiwan) was used to extract genomic DNA from the pure fungal mycelia grown for a week on PDA, following the manufacturer’s instructions. The internal transcribed spacer (ITS), actin (*act*), beta-tubulin (*tub*), calmodulin (*cal*), glyceraldehyde-3-phosphate dehydrogenase (*gapdh*), RNA polymerase II second largest subunit (*rpb2*), and translation elongation factor 1-alpha (*tef1-α*) genes were carried out using the primer pairs ITS5/ITS4, ACT512F/ACT738R, T1/T2 or T1/T22, CL1C/CL2C or CAL-228F/CAL-737R, GDF1/GPDHR2, RPB2-5F2/RPB2-7cR, and EF1/EF2 or EF1-728F/EF1-986R, following the previous studies (Table 1) [30,32,33,34,35,36,37]. The PCR products were purified and sequenced by 1st Base Co., Ltd., Selangor, Malaysia.

#### 2.3.3. Sequence Alignment and Phylogenetic Analyses

The BLAST algorithm available through the National Center for Biotechnology Information portal (http://blast.ncbi.nlm.nih.gov, accessed on 25 May 2025) was utilized to conduct similarity searches. The sequences from this study, along with those obtained from previous studies and the GenBank database, were selected for phylogenetic analyses. Sequence alignment was performed using the MUSCLE [38], followed by manual refinements in BioEdit 6.0.7 [39]. Phylogenetic relationships were constructed through a maximum likelihood (ML) methodology on the GTRCAT model using RAxML-HPC2 version 8.2.12, accessed via the CIPRES web platform, with 1000 bootstrap iterations providing statistical support [40,41]. The resulting phylogenetic trees were then visually rendered using FigTree v1.4.0 software [42].

### 2.4. Pathogenicity Tests

Healthy commercial culinary melons were surface sterilized by soaking in a diluted 1.5% (*v*/*v*) aqueous solution prepared from commercial 10% (*v*/*v*) sodium hypochlorite for 5 min and then rinsed three times with sterile distilled water. Following surface disinfection, fruits were then allowed to air-dry for 10 min at room temperature (25 ± 2 °C). After air-drying, a uniform wound (5 pores, each 1 cm deep and 1 mm wide) was made at the equator of each fruit using aseptic needles following the method described in previous studies [5,7]. Eight fungal strains (SDBR-CMU540 to SDBR-CMU547) were obtained and used in this experiment. The conidia from each fungal isolate cultivated for two weeks on PDA were collected and used in this experiment. The conidia were suspended in sterile distilled water and adjusted to a concentration of 1 × 10^6^ conidia/mL using a hemocytometer [7,43]. Subsequently, a conidial suspension (500 µL) of each fungal isolate was separately dropped onto the wounded fruits, while sterile distilled water was used to inoculate the wounded fruits as a control. Then, the inoculated fruit was stored in an individual sterile plastic container (26 cm × 35.5 cm × 20 cm) under 80% relative humidity conditions. The plastic containers were maintained in a growth chamber at a temperature of 25 °C under a 12 h light cycle for a duration of one week. Each treatment was carried out with 10 replications, performed independently twice under the same conditions. The level of disease infections was evaluated using a score of 1–25% (mild), 26–50% (moderate), 51–75% (severe), and 76–100% (very severe) based on the extent of disease infection on the damaged portions of the fruit [44]. Koch’s postulates were confirmed by re-isolating the fungi from the inoculated fruits and identified by morphological characteristics and molecular data.

### 2.5. Commercial Fungicide Sensitivity Tests

A total of eleven commercially fungicides, including azoxystrobin (Amistar^®^, Samut Prakan, Thailand), benalaxyl-M (4%) + mancozeb (65%) (Fantic M WG^®^, Bangkok, Thailand), captan (Captan 50^®^, Bangkok, Thailand), carbendazim (Dazine^®^, Bangkok, Thailand), copper hydroxide (Funguran-OH^®^, Ratchaburi, Thailand), copper oxychloride (Copina 85 WP^®^, Bangkok, Thailand), cyproconazole (Alto^®^, Samut Prakan, Thailand), difenoconazole (Score^®^, Samut Prakan, Thailand), difenoconazole (12.5%) + azoxystrobin (20%) (Ortiva^®^, Samut Prakan, Thailand), mancozeb (Newthane M-80^®^, Bangkok, Thailand), and propineb (Antracol^®^, Samut Prakan, Thailand), were examined in this study according to the method described by Suwannarach et al. [7]. All fungicides used in this study are approved for use and commercially available in Thailand. Each fungicide was applied according to the manufacturer’s recommended concentrations and application guidelines. According to the guidelines for each fungicide, the recommended dosages used in this study were as follows: azoxystrobin (125 ppm), benalaxyl-M (4%) + mancozeb (65%) (80 + 1300 ppm), captan (1000 ppm), carbendazim (750 ppm), copper hydroxide (1540 ppm), copper oxychloride (1700 ppm), cyproconazole (60 ppm), difenoconazole (187 ppm), difenoconazole (12.5%) + azoxystrobin (20%) (240 + 385 ppm), mancozeb (1300 ppm), and propineb (1400 ppm). The final concentrations of the recommended dosages were prepared by dissolving each fungicide in autoclaved PDA. A mycelial plug (5 mm in diameter) from a colony grown on PDA for one week of each fungal strain was inoculated onto the tested ager media. The control treatment did not receive any fungicide treatment. The plates were incubated in darkness at 25 °C for 7 to 14 days, or until the control plates were fully covered with fungal mycelia. The colony diameter of each strain was measured, and the percentage growth inhibition was calculated using the formula provided by Pandey et al. [45]. Based on growth inhibition, each fungal strain was classified as sensitive (≥50%), insensitive (<50%), or fully inhibited (100%) [7,45]. For each fungicide and fungal strain, five replications were performed. The experiments were repeated independently twice under the same conditions.

### 2.6. Statistical Analysis

SPSS software version 26 was used. The Shapiro–Wilk test was applied to examine data from the two repeated fungicide sensitivity experiments at a significance level of *p* < 0.05 to test for normality. The data from these repeated experiments were evaluated for the assumptions of one-way analysis of variance (ANOVA), as the results showed non-significant findings. Significant differences were then determined at *p* < 0.05 using Duncan’s Multiple Range Test (DMRT).

## 3. Results

### 3.1. Disease Symptoms

**Figure 1:**
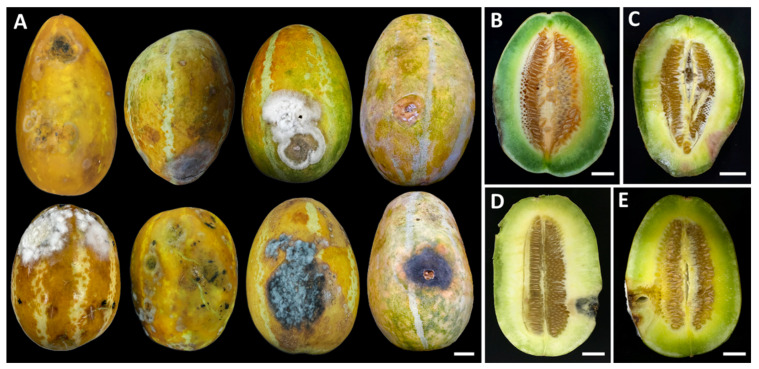
Symptoms of diseases in culinary melon fruits during the postharvest storage period (**A**). Longitudinal sections of anthracnose (**B**,**C**), charcoal rot (**D**), and rot (**E**) symptoms. Scale bars = 20 mm.

Three distinct postharvest diseases were observed in culinary melons (Figure 1), with anthracnose identified as the first. The symptoms of anthracnose typically appeared as small, water-soaked lesions on the fruit surface. Over time, these spots expanded, becoming depressed and dark brown to black, frequently exhibiting concentric ring patterns. In advanced stages, the lesions could coalesce, leading to extensive fruit rotting. Under humid conditions, pink to orange spore masses were developed on lesions. The second was charcoal rot. Charcoal rot was presented by dry, sunken, and dark brown to black lesions on the fruit surface. Infected areas became firm, often accompanied by cracking or shriveling of the rind. As the disease progressed, a distinctive gray to black discoloration could be visible beneath the epidermis. Black microsclerotia were embedded in the affected tissues. The last was fruit rot. The initial symptoms appeared as small, water-soaked or pale brown spots on the fruit surface. The spots enlarged and became irregularly shaped lesions. As the disease progressed, the lesions became soft and water-soaked, and the fruit eventually decayed and rotted completely. White and gray mycelia covered the decaying lesions.

### 3.2. Fungal Isolation and Morphological Study

Eight fungal strains were isolated, and their pure cultures were deposited in the SDBR Laboratory Culture Collection (numbered SDBR-CMU540 to SDBR-CMU547). Three strains (SDBR-CMU540 to SDBR-CMU542) were isolated from anthracnose disease samples, while the other three (SDBR-CMU543 to SDBR-CMU545) were obtained from charcoal rot. The remaining two strains (SDBR-CMU546 and SDBR-CMU547) were isolated from fruit rot. Morphological examination revealed that three strains (SDBR-CMU540 to SDBR-CMU542) belonged to the genus *Colletotrichum*, three (SDBR-CMU543 to SDBR-CMU545) to the genus *Macrophomina*, and two (SDBR-CMU546 and SDBR-CMU547) to the genus *Fusarium*. Subsequently, multi-gene molecular phylogeny was employed for their species identification.

### 3.3. Phylogenetic Analysis

The DNA sequences from each fungal isolate were submitted to the GenBank database, and the BLAST search results are presented in Table 2.

**Figure 2:**
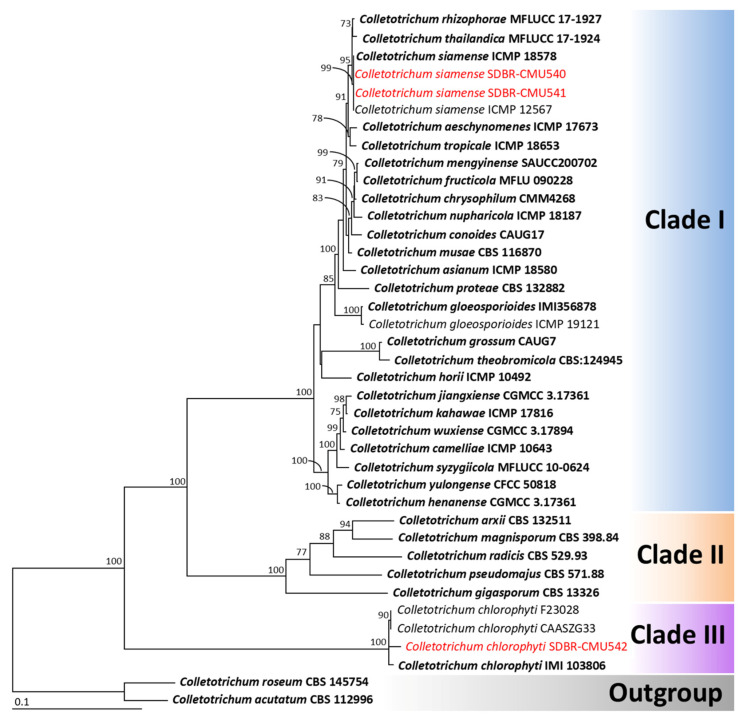
Phylogram derived from maximum likelihood analysis of 39 fungal strains of the combined ITS, *gapdh*, *cal*, *act*, and *tub* sequences. *Colletotrichum acutatum* CBS 112996 and *C. roseum* CBS 145754 were set as the outgroup. The numbers above branches represent bootstrap percentages, and values >70% are shown. The scale bar represents the expected number of nucleotide substitutions per site. The fungal strain obtained from this study is in red. Ex-type strains are bold.

The phylogenetic reconstruction of the *Colletotrichum* species was performed using a multi-gene approach incorporating five loci (ITS, *gapdh*, *cal*, *act*, and *tub*), in accordance with established identification protocols from previous studies [32,33,35]. The aligned data set contained 2775 bp including gaps (ITS: 1–622, *gapdh*: 623–928, *cal*: 929–1711, *tub*: 1712–1998, *act*: 1999–2775), with 39 taxa. The outgroup comprised *C. acutatum* and *C. roseum*, both belonging to the *C. acutatum* species complex. The alignment has 1231 distinct alignment patterns, with 20.02% of undetermined characters or gaps. The RAxML analysis resulted in a final ML optimization likelihood value of -13636.9451. A phylogenetic tree is represented in Figure 2. Three clades were identified as Clade I (*C. gloeosporioides* species complex), Clade II (*C. gigasporum* species complex), and Clade III (*C. chlorophyti*). The phylogenetic tree successfully assigned two fungal strains, SDBR-CMU540 and SDBR-CMU541, within the *C. gloeosporioides* species complex and the clade of *C. siamense*, which included the ex-type strain ICMP 18578. This clade showed 99% bootstrap (BS) support. Meanwhile, strain SDBR-CMU540 belonged to *C. chlorophyti*, which included the ex-type strain IMI 103806 with 100% bootstrap support for this clade. Therefore, strains SDBR-CMU540 and SDBR-CMU541 were identified as *C. siamense* and SDBR-CMU543 was identified as *C. chlorophyti*, with their morphological characteristics described below.

**Figure 3:**
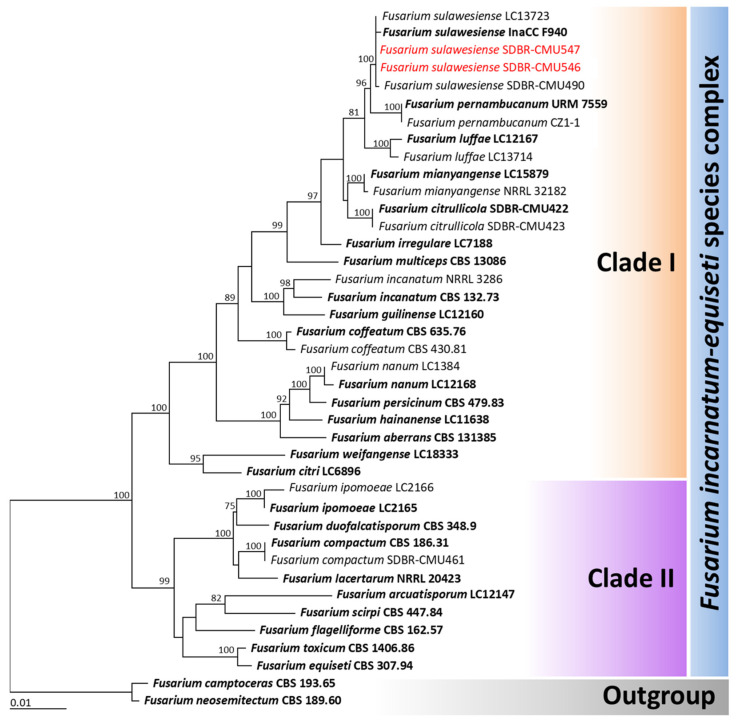
Phylogram derived from maximum likelihood analysis of 40 fungal strains of the combined ITS, *act*, *cal*, and *tef1-α* sequences. *Fusarium camptoceras* CBS 193.65 and *F. neosemitectum* CBS 189.60 were set as the outgroup. The numbers above branches represent bootstrap percentages, and values >70% are shown. The scale bar represents the expected number of nucleotide substitutions per site. The fungal strain obtained from this study is in red. Ex-type strains are bold.

Based on BLAST results, strains SDBR-CMU546 and SDBR-CMU547 belonged to the *Fusarium incarnatum-equiseti* species complex. For the phylogenetic analysis of species within this complex, three genes, *tef1-α*, *cal*, and *rpb2* were used, following previous phylogenetic studies [31,34,46]. The aligned data set contained 2093 bp including gaps (*tef1-α*: 1–638, *cal*: 639–1217, *rpb2*: 1218–2093), with 40 taxa. *Fusarium camptoceras* and *F. neosemitectum* from the *F. camptoceras* species complex were designated as the outgroup. The alignment has 392 distinct alignment patterns, with 3.19% of undetermined characters or gaps. The RAxML analysis resulted in a final ML optimization likelihood value of −6982.5524. The phylogenetic tree is displayed in Figure 3. Two main clades of the *F. incarnatum-equiseti* species complex were assigned as Incarnatum clade (clade I) and Equiseti clade (clade II). The results revealed that strains SDBR-CMU546 and SDBR-CMU547 belonged to the Incarnatum clade within *F. sulawesiense*, which included the ex-type strain InaCC F940 (100% BS subport). Consequently, both fungal strains were determined to be *F. sulawesiense*, with their morphological characteristics detailed below.

**Figure 4:**
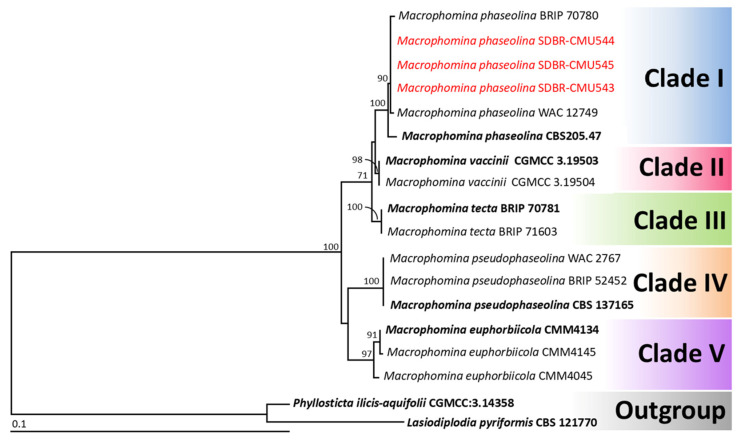
Phylogram derived from maximum likelihood analysis of 18 fungal strains of the combined ITS, *act*, *cal*, and *tef1-α* sequences. *Lasiodiplodia pyriformis* CBS 121770 and *Phyllosticta ilicis-aquifolii* CGMCC:3.14358 were set as the outgroup. The numbers above branches represent bootstrap percentages, and values > 70% are shown. The scale bar represents the expected number of nucleotide substitutions per site. The fungal strain obtained from this study is in red. Ex-type strains are bold.

Phylogenetic analysis of *Macrophomina* species was conducted using a combined four-gene data set comprising ITS, *act*, *cal*, and *tef1-α* loci, following standard identification methodologies established in previous research [36,37]. The aligned data set contained 1760 bp including gaps (ITS: 1–626, *act*: 627–895, *cal*: 896–1459, *tef1-α*: 1460–1760), with 18 taxa. *Lasiodiplodia pyriformis* and *Phyllosticta ilicis-aquifolii* were designated as the outgroup. The alignment has 161 distinct alignment patterns, with 16.55% of undetermined characters or gaps. The RAxML analysis resulted in a final ML optimization likelihood value of −3475.9940. A phylogenetic tree is shown in Figure 4. Five main clades of *Macrophomina* species were assigned as *M. phaseolina* (clade I), *M. vaccinii* (clade II), *M. tecta* (clade III), *M. pseudophaseolina* (clade IV), and *M. euphorbiicola* (clade V), according to the findings of previous phylogenetic studies [36,37]. The phylogenetic tree successfully placed three fungal strains (SDBR-CMU543, SDBR-CMU544, and SDBR-CMU545) within *M. phaseolina*, with this clade receiving 99% BS support. Therefore, these three strains were identified as *M. phaseolina*, and their morphological descriptions are provided below.

### 3.4. Morphological Description

#### 3.4.1. *Colletotrichum siamense*

**Figure 5:**
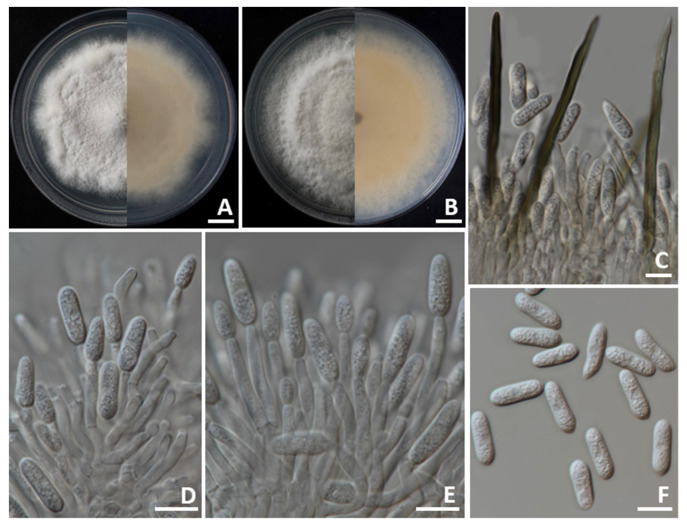
*Colletotrichum siamense*. Colony morphology of strains SDBR-CMU540 (**A**) and SDBR-CMU541 (**B**) grown on potato dextrose agar after one week of incubation at 25 °C (left, surface view and right, reverse view). Micromorphology of strains SDBR-CMU540 (**C**–**F**). Setae and conidiogenous cells giving rise to conidia (**C**). Conidiogenous cells giving rise to conidia (**D**,**E**). Conidia (**F**). Scale bars: (**A**,**B**) = 10 mm; (**C**–**F**) = 10 µm.

Colonies on PDA achieved diameters between 70 and 85 mm when incubated for 7 days at 25 °C in darkness (Figure 5). The colonies exhibited a cottony texture with circular growth and wavy edges. Initially, the surface appeared pale gray centrally with white margins, gradually turning gray as the culture aged. The reverse side displayed colors varying from pale orange to pale brown. Conidial masses were present, showing light orange to orange. Setae displayed brown to dark brown pigmentation, possessed smooth walls with 2–4 septations, reached lengths of 70–125 µm (*n* = 50), with tips that were either acute or obtuse. Appressoria were not observed. Conidiophores exhibited hyaline, septation, branching, and ranged from clavate to cylindrical in form. Conidiogenous cells displayed hyaline, clavate to cylindrical, and measured 15–20 × 3–5 µm (*n* = 50). The conidia appeared as smooth-walled, hyaline, aseptate, subcylindrical to oblong, rounded tips, guttulate, and sized 10–20 × 4–5 µm (*n* = 50). Both *C. siamense* strains SDBR-CMU540 and SDBR-CMU541 obtained in this study showed morphological characteristics that corresponded with previous descriptions of the species [47,48].

#### 3.4.2. *Colletotrichum chlorophyti*

**Figure 6:**
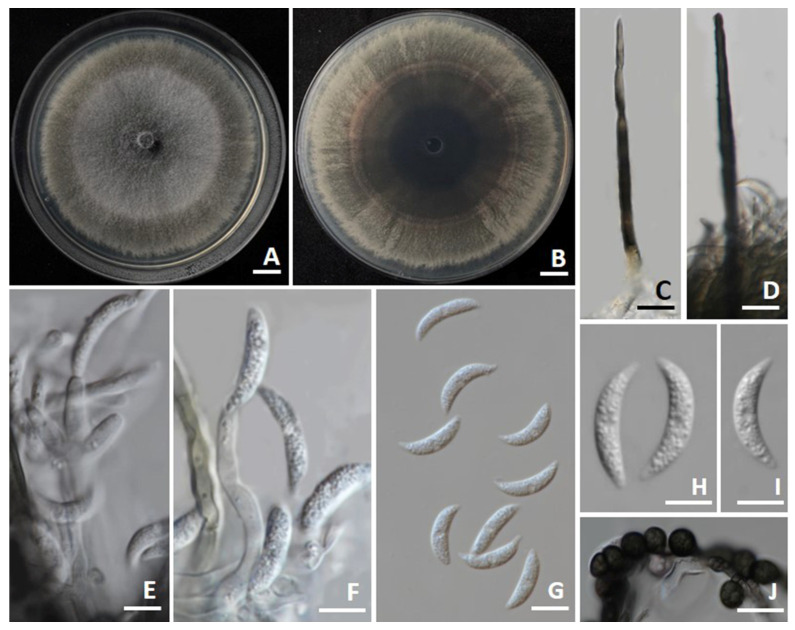
*Colletotrichum chlorophyti*. Colony morphology of strains SDBR-CMU542 grown on potato dextrose agar after one week of incubation at 25 °C. Surface view (**A**) and reverse view (**B**). Micromorphology of strains SDBR-CMU542 (**C**–**J**). Setae (**C**,**D**). Conidiogenous cells giving rise to conidia (**E**,**F**). Conidia (**G**–**I**). Chlamydospores (**J**) Scale bars: (**A**,**B**) = 10 mm; (**C**–**J**) = 10 µm.

The colonies on PDA achieved diameters between 75 and 85 mm when incubated for 7 days at 25 °C in darkness (Figure 6). The colonies exhibited a flat, slimy surface with olivaceous-gray to grayish-black toward the margins. The reverse side appeared as pale olivaceous-gray to grayish-black. Conidial masses were present, showing up as light orange. Setae were brown to dark brown, smooth-walled, with 2–4 septa, measuring 80–120 µm in length (*n* = 50), and had tips that ranged from acute to finely verruculose. Appressoria were not observed. Conidiophores were hyaline, septate, occasionally branched, and ranged from clavate to cylindrical in shape. Conidiogenous cells displayed hyaline, ampulliform to elongate ampulliform, and measured 8–25 × 3–5 µm (*n* = 50). Conidia appeared as smooth-walled, hyaline, aseptate, curved, base truncate, apex acute, more tapered and stronger curved than base, guttulate, and sized 15–25 × 3–5 µm (*n* = 50). Chlamydospores were dark brown to black, globose to subglobose, thick-walled, and occurred in clusters or chains, measuring 6–10 µm in diameter. The morphological characteristics of *C. chlorophyti* SDBR-CMU542 showed similarity to the morphological description of *C. chlorophyti* in the publication by Damm et al. [49].

#### 3.4.3. *Fusarium sulawesiense*

**Figure 7:**
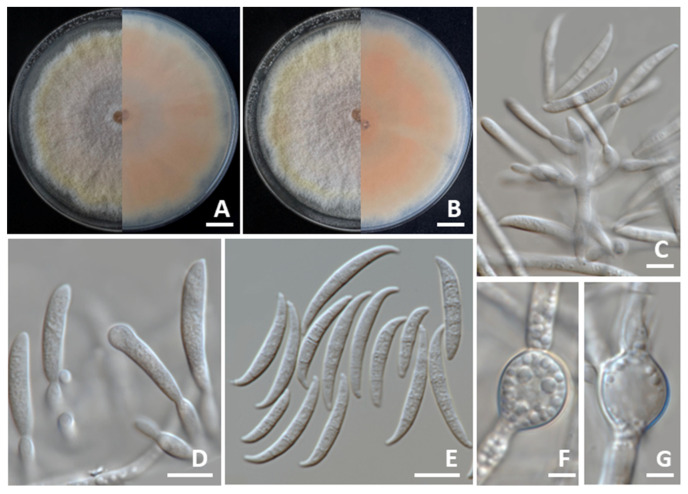
*Fusarium sulawesiense*. Colony morphology of strains SDBR-CMU546 (**A**) and SDBR-CMU547 (**B**) grown on potato dextrose agar after one week of incubation at 25 °C (left, surface view and right, reverse view). Aerial conidiophores (**C**,**D**). Conidia (**E**). Chlamydospores (**F**,**G**). Scale bars: (**A**,**B**) = 10 mm; (**C**–**E**) = 10 µm; (**F**,**G**) = 5 µm.

Colonies on PDA reached diameters of 80–85 mm after 7 days of incubation at 25 °C in darkness (Figure 7). The colonies exhibited a greyish-yellow center that gradually transitioned to orange white toward the margins. They were elevated with smooth, entire edges. The reverse side appeared light yellow. Sporodochia were absent. Conidiophores developed on the aerial mycelium, irregularly or verticillately branched, and measured 8.5–118.5 μm. Phialides were both mono- and polyphialidic, subulate to subcylindrical in shape, smooth, thin-walled, and measured 8.5–25.5 × 2.0–5.0 μm. Chlamydospores were hyaline to pale yellow with age, globose to ellipsoidal, smooth-walled, and occurred intercalarily or terminally, either solitary or in long chains, measuring 10.5–26.5 × 5.0–20.5 μm. Conidia were falcate, hyaline, smooth, thin-walled, unequally curved, with a pointed to blunt apical cell, a poorly developed foot-shaped basal cell, 3–8-septate, and measured 18.5–55.0 × 3.0–6.0 μm. The morphological characteristics of *F. sulawesiense* strains SDBR-CMU546 and SDBR-CMU547 identified in this study closely matched the descriptions provided by Maryani et al. [50] and Yi et al. [51].

#### 3.4.4. *Macrophomina phaseolina*

**Figure 8:**
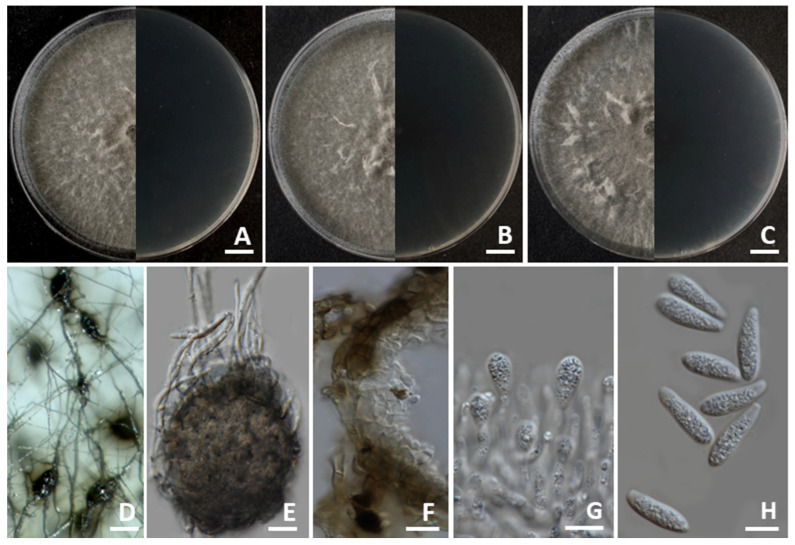
*Macrophomina phaseolina*. Colony morphology of strains SDBR-CMU543 (**A**), SDBR-CMU544 (**B**), and SDBR-CMU545 (**C**) grown on potato dextrose agar after one week of incubation at 25 °C (left, surface view and right, reverse view). Micromorphology of strains SDBR-CMU543 (**D**–**H**). Microsclerotia (**D**). Pycnidia (**E**). Pycnidia wall (**F**). Conidiogenous cells giving rise to conidia (**G**). Conidia (**H**). Scale bars: (**A**–**C**) = 10 mm; (**D**) = 100 µm; (**E**) = 25 µm; (**F**) = 20 µm; (**G**,**H**) = 10 µm.

Colonies on PDA achieved diameters between 80 and 85 mm when incubated for 7 days at 25 °C in darkness (Figure 8). The colonies exhibited fluffy aerial mycelia with a buff-colored appearance, turning vinaceous buff to pale olivaceous grey, and then becoming dark gray with age. The reverse side appeared as olivaceous gray to black. Microsclerotia formed from intertwined primary and secondary hyphae, dark brown to black, spherical, oval to oblong or irregular, and measured 80–250 μm in diameter. Pycnidia formed after three weeks of incubation (25 °C with 12 h photoperiod), were black, globose (180–200 μm in diameter), and with an apical ostiole (20–30 μm). Conidiophores were present inside the pycnidium, hyaline, cylindrical, and measured 15–20 × 3–6 μm. Conidia were ellipsoid to obovoid and averaged 12.0–25.0 × 7.0–11.0 μm. Immature conidia possessed apical mucoid appendages. The morphological characteristics of *M. phaseolina* SDBR-CMU543, SDBR-CMU544, and SDBR-CMU545 showed similarity to the morphological description of *M. phaseolina* in previous publications [18,19,36,52].

### 3.5. Pathogenicity Test

**Figure 9:**
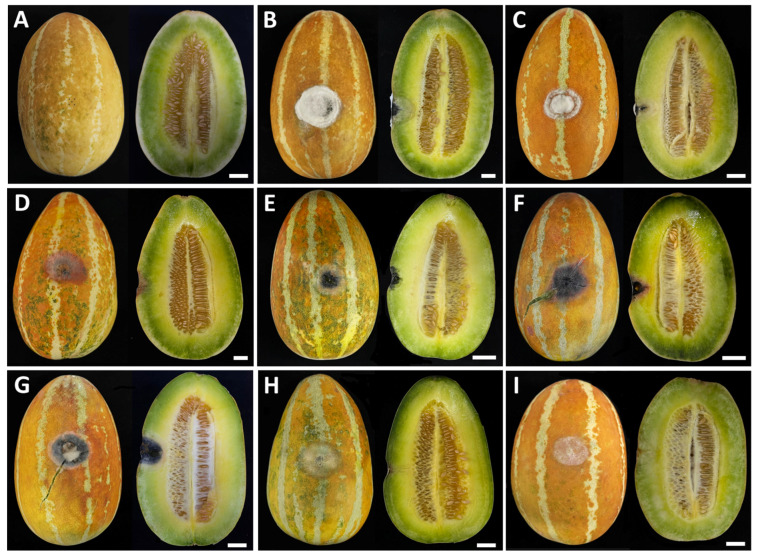
Pathogenicity test of fungal causal agents of postharvest diseases on culinary melons after 7 days of inoculation (left, top view, and right, longitudinal sections). Control fruit inoculated with sterile water (**A**). Disease symptoms after inoculation with *C. siamense* SDBR-CMU540 (**B**), *C. siamense* SDBR-CMU541 (**C**), *C. chlorophyti* SDBR-CMU542 (**D**), *M. phaseolina* SDBR-CMU543 (**E**), *M. phaseolina* SDBR-CMU544 (**F**), *M. phaseolina* SDBR-CMU545 (**G**), *F. sulawesiense* SDBR-CMU546 (**H**), and *F. sulawesiense* SDBR-CMU547 (**I**). Scale bars = 20 mm.

The pathogenicity of each fungal strain was confirmed by observing symptoms in inoculated fruits and comparing them with the control treatment. All fungal strains were found to be pathogenic to culinary melon fruits inoculated, while the control fruits remained asymptomatic (Figure 9A). Three days of post-inoculation, the first symptoms were visible on the culinary melon fruits, which exhibited spots and lesions. By the seventh day post-inoculation, culinary melons infected with the fungal pathogen causing anthracnose (*C. siamense* strains SDBR-CMU540 and SDBR-CMU541, and *C. chlorophyti* strain SDBR-CMU542) developed expanding yellowish-brown to light brown water-soaked lesions surrounded by white mycelia (Figure 9B–D), showing mild infection with disease scores of 5–20%. The longitudinal sections revealed decomposing internal tissue encircled by water-soaked areas at the inoculation sites. Within 14 days, the lesions expanded and became necrotic, advancing to moderate–severe infections (disease scores of 50–70%). After three weeks, infections reached very severe levels (80–85% disease scores), ultimately resulting in completely softened and rotted fruits.

The culinary melon fruits inoculated with the fungal pathogen causing charcoal rot (*M. phaseolina* strains SDBR-CMU543 to SDBR-CMU545) developed expanding sunken, and dark brown to black lesions, some fruits were cracking, and white to gray mycelia surrounded lesions (Figure 9E–G), showing mild infection with disease scores of 5–25%. The longitudinal sections revealed decomposing internal tissue, surrounded by black lesions and water-soaked areas at the inoculation sites. Within 14 days, the lesions expanded and became necrotic, advancing to moderate–severe infections (disease scores of 60–70%). The disease symptoms were very severe at 80–90% disease scores and completely rotted within three weeks.

After seven days, culinary melon fruits developed yellowish-brown to light brown and water-soaked areas after inoculation with the fungal pathogen causing rot (*F. sulawesiense* strains SDBR-CMU546 and SDBR-CMU547), white mycelia surrounded lesions, and mild infection was shown with disease scores of 20–25% (Figure 9H,I). Longitudinal sections demonstrated the presence of decaying internal tissue and water-soaked areas at the inoculation sites. Lesions showed expansion and moderate to severe infections, with disease scores ranging from 70% to 75%. This condition progressed to extreme severity, with disease scores between 80% and 95%, ultimately resulting in the complete rotting of the fruits within three weeks.

The symptoms of anthracnose, charcoal rot, and fruit rot observed in laboratory experiments closely resembled those encountered during the postharvest storage phase. The fungi isolated from each inoculated fruit were successfully re-isolated through cultivation on PDA, thereby fulfilling Koch’s postulates. Based on morphological characterization, the re-isolated fungi associated with anthracnose, charcoal rot, and fruit rot lesions were identified as *C. siamense* and *C. chlorophyti*, *M. phaseolina*, and *F. sulawesiense*, respectively.

### 3.6. Response of Fungal Pathogens to Commercial Fungicides

**Figure 10:**
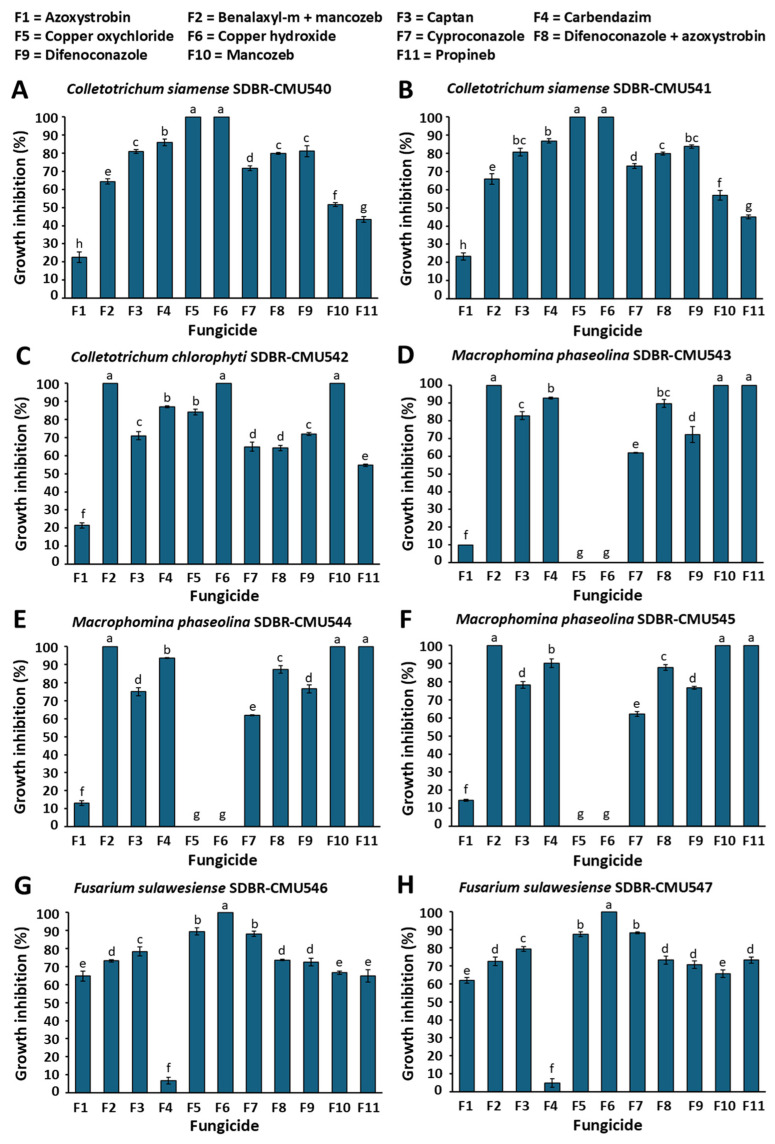
The percentage of mycelial growth inhibition in *C. siamense* (**A**,**B**), *C. chlorophyti* (**C**), *M. phaseolina* (**D**–**F**), and *F. sulawesiense* (**G**,**H**) at the recommended dosage of each fungicide and classified as totally inhibited (100% inhibition), sensitive (≥50% inhibition), or insensitive (<50% inhibition). Data are displayed as means, with the error bars on each graph representing the ± standard deviation. Distinct letters assigned to each graph for the same fungal strain denote a statistically significant difference (*p* < 0.05), as determined by the DMRT.

Fungal pathogen sensitivity to commercial fungicides was evaluated by measuring mycelial growth inhibition at each fungicide’s recommended dosage. Responses were categorized as completely inhibited (100% inhibition), sensitive (≥50% inhibition), or insensitive (<50% inhibition). The findings revealed that mycelial growth inhibition varied based on the fungicides used, fungal species, and strains, as illustrated in Figure 10. The Shapiro–Wilk test confirmed that mycelial inhibition percentages for each fungal strain across different fungicides followed a normal distribution (*p* < 0.001). To determine statistically significant differences, we performed an analysis of variance (ANOVA), followed by Duncan’s Multiple Range Test (DMRT) at a significance level of *p* < 0.05. Among the 11 fungicides evaluated, copper oxychloride and copper hydroxide completely inhibited (100%) the growth of *C. siamense*. Both *C. siamense* strains were sensitive to benalaxyl-M + mancozeb, captan, carbendazim, cyproconazole, difenoconazole, difenoconazole + azoxystrobin, and mancozeb, while they showed resistance to azoxystrobin and propineb. For *C. chlorophyti*, benalaxyl-M + mancozeb, copper hydroxide, and mancozeb achieved complete (100%) inhibition, while the fungus showed insensitivity to azoxystrobin. All *M. phaseolina* strains were completely inhibited (100%) by benalaxyl-M + mancozeb, mancozeb, and propineb, but were insensitive to azoxystrobin. Copper oxychloride and copper hydroxide had no effect on the growth of *M. phaseolina*. In both *F. sulawesiense* strains, only copper hydroxide achieved complete inhibition (100%) of growth. They showed sensitivity to all other nine tested fungicides, with the exception of carbendazim.

## 4. Discussion

Fungal pathogens are among the most significant contributors to postharvest losses in cucurbit crops, including culinary melon, cantaloupe, and muskmelon, severely affecting fruit quality and reducing commercial marketability [3,53,54]. Several fungal genera, including *Alternaria*, *Colletotrichum*, *Diaporthe*, *Fusarium*, *Lasiodiplodia*, *Macrophomina*, *Neoscytalidium*, *Penicillium*, *Sclerotium*, and *Stagonosporopsis*, are widely recognized as common postharvest pathogens of cucurbit crops and have been reported across various melon-producing regions worldwide [4,7,8,9,10,11,12,13,14,15]. Specifically, several *Colletotrichum* and *Fusarium* species are responsible for anthracnose and fruit rot, respectively [5,7,28]. Additionally, *Macrophomina* species are responsible for charcoal rot in these crops [3,21,22]. In this investigation, culinary melons collected during the postharvest storage in northern Thailand (2023–2024) exhibited anthracnose symptoms from which two *C. siamense* strains and one *C. chlorophyti* strain were isolated. One *F. sulawesiense* strain was derived from fruit rot lesions, and three *M. phaseolina* strains were obtained from charcoal rot lesions. All obtained fungal strains were identified through morphological characterization and multi-gene phylogenetic analysis, following the protocols established in previous studies [30,32,33,34,35,36,37]. Koch’s postulates were confirmed by conducting pathogenicity tests on all isolated strains of *C. chlorophyti*, *C. siamense*, *F. sulawesiense*, and *M. phaseolina*, thus verifying that they are the causal agents of the postharvest disease symptoms affecting the culinary melons observed in the field survey. However, future investigations should employ non-wound inoculation techniques to more accurately mimic natural infection conditions and thus provide a more thorough understanding of pathogen dynamics under postharvest conditions.

*Colletotrichum* is responsible for causing serious diseases in numerous important fruit crops cultivated globally [55,56,57,58]. Prior to this study, *C. aenigma*, *C. chlorophyti*, *C. fructicola*, and *C. orbiculare* had been reported as causal agents of anthracnose on culinary melon fruits in Japan [3,59]. *Colletotrichum truncatum*, the causal agents of anthracnose on culinary melon fruits, has been reported in China [22]. Moreover, *C. chlorophyti* has also been associated with anthracnose disease of watermelon in China [21]. *Colletotrichum siamense* has been reported as a causal agent of anthracnose in several fruit crops, including avocado, mango, papaya, and strawberry among others [55,56,57,58,60], but has not been reported in culinary melon fruit. Therefore, this study represents the first report of *C. siamense* causing anthracnose on culinary melon fruit worldwide, and the first documented occurrence of *C. chlorophyti* as a causal agent of anthracnose disease in culinary melon fruits in Thailand.

*Fusarium* is responsible for fruit rot in cucurbit crops worldwide. Several species belonging to the *F. incarnatum-equiseti* species complex have been reported as pathogens causing fruit rot in cantaloupe, melons, and muskmelons. *Fusarium equiseti* caused fruit rot on cantaloupe and oriental melon fruits in China [61], Korea [62], and Thailand [28]. *Fusarium incarnatum* has been documented as causing postharvest fruit rot in muskmelons in Thailand [29] and in oriental melons in Korea [62]. Muskmelon fruit rot caused by *F. compactum*, *F. jinanense*, *F. melonis*, *mianyangense*, and *F. sulawesiense* has been reported in Thailand [5,7]. In Brazil, *F. sulawesiense* and *F. jinanense* has been associated with fruit rot in canary melon, while *F. pernambucanum* has been identified as the causal agent of fruit rot in both Santa Claus melon and Galia melon [4,63,64,65,66]. In China, *F. incarnatum*, *F. luffae*, *F. nanum*, *F. pernambucanum*, and *F. sulawesiense* have been identified as pathogens responsible for fruit rot in muskmelons [34,67,68,69,70,71]. Additionally, fruit rot in muskmelons has been associated with various *Fusarium* species from various species complexes. These include members of the *F. fujikuroi* species complex (e.g., *F. annulatum*, *F. moniliforme*, and *F. proliferatum*), the *F. oxysporum* species complex (such as *F. kalimantanense* and *F. oxysporum*), the *F. sambucinum* species complex (including *F. asiaticum*, *F. graminearum*, and *F. sambucinum*), and the *F. solani* species complex (e.g., *F. falciforme* and *F. solani*) [61,62,64,71,72,73]. Recently, only *F. ipomoeae* has been reported to cause fruit rot in culinary melon fruit in China [74]. Therefore, this study represents the first report of *F. sulawesiense* causing fruit rot on culinary melon fruit worldwide.

*Macrophomina phaseolina* is a fungal pathogen that causes charcoal rot in both seedlings and mature plants of over 500 plant species worldwide, including cantaloupe [20], culinary melon [18], watermelon [20,75,76], and other melons [18,75]. This pathogen has been documented as causing charcoal rot in cantaloupe (*C. melo* var. *cantalupo*) and melon fruits (*C. melo*) [77], but had not been previously reported in culinary melon fruit. Consequently, this study represents the first global report of *M. phaseolina* as a causal agent of charcoal rot in culinary melon fruit.

The efficacy of various commercial fungicides was evaluated against the isolated fungal pathogens to determine their sensitivity for disease management. In laboratory experiments, fungicides can effectively suppress the mycelial growth of plant pathogenic fungi, resulting in varying degrees of insensitivity (resistance), sensitivity, and inhibition [44,78,79]. In this study, notable differences in inhibition, sensitivity, and resistance to fungicides were observed across various fungicide types, fungal species, and individual strains. These findings are consistent with previous research, which has shown that the efficacy of fungicidal treatments is influenced not only by the type and concentration of fungicide but also by the variability among fungal species and strains [7,44,79]. In the fungicide screening test conducted in this study, copper oxychloride and copper hydroxide achieved the complete inhibition of *C. siamense*. Benalaxyl-M + mancozeb, copper hydroxide, and mancozeb demonstrated the complete inhibition of *C. chlorophyti*. These copper-based fungicides showed particularly strong efficacy against both *Colletotrichum* species, suggesting their potential as effective control agents for anthracnose management in culinary melon. *Colletotrichum siamense* and *C. chlorophyti* associated with culinary melon anthracnose were resistant to azoxystrobin, while *C. siamense* was also resistant to propineb. These findings are consistent with the azoxystrobin resistance reported in *C. siamense* strains causing mango anthracnose in Thailand [79]. However, *C. siamense* strains isolated from orange anthracnose were found to be sensitive to azoxystrobin [80]. In addition, Apithanasakulngeon et al. [81] also found that *C. siamense* strains causing durian anthracnose in Thailand were sensitive to difenoconazole, mancozeb, and prochloraz, but they were resistant to chlorothalonil and pyraclostrobin. *Colletotrichum siamense* strains, which cause peach and walnut anthracnoses in China, were highly sensitive to prochloraz [82,83].

In this study, among the *F. sulawesiense* strains tested, only copper hydroxide achieved complete inhibition, while the strains exhibited resistance to carbendazim. This suggests that copper hydroxide has potential as an effective control agent for managing the fruit rot caused by *F. sulawesiense* in culinary melon. However, *F. sulawesiense* strains causing fruit rot in muskmelon in Thailand showed sensitivity only to copper oxychloride, along with *F. compactum*, *F. jinanense*, and *F. mianyangense* [7]. The experiment conducted by Maniçoba et al. [84] revealed that *Fusarium* pathogens (*F. falciforme*, *F. kalimantanense*, *F. pernambucanum*, and *F. sulawesiense*) associated with melon fruit rot in Brazil were sensitive to azoxystrobin + fludioxonil and imazalil. Thus, determining the in vitro sensitivity and resistance profiles of fungicides against the *Fusarium* species causing muskmelon fruit rot would provide valuable insights for in vivo applications and disease management strategies in Thailand and worldwide. The complete growth inhibition (100%) of all *M. phaseolina* strains was achieved with benalaxyl-M + mancozeb, mancozeb, and propineb, whereas these strains demonstrated resistance to azoxystrobin, copper oxychloride, and copper hydroxide. This suggests that benalaxyl-M + mancozeb, mancozeb, and propineb have potential as effective control agents for charcoal rot management in culinary melon. Several previous studies have evaluated various fungicides against *M. phaseolina*, including carbendazim, difenoconazole, benomyl, azoxystrobin, and dazomet at different concentrations in both in vitro and in vivo assays [85,86,87,88,89]. These studies demonstrated that *M. phaseolina* exhibits high sensitivity to carbendazim [89], and field applications of carbendazim significantly reduced disease incidence while improving plant survival rates [90]. Nevertheless, in vitro fungicide sensitivity results may not accurately reflect in vivo efficacy, as environmental conditions, plant–pathogen interactions, and fungicide metabolism within plant tissues can significantly influence treatment outcomes. Consequently, follow-up in vivo studies are essential to confirm the practical applicability of these in vitro sensitivity profiles. Although this study assessed fungicide sensitivity based on the mycelial growth inhibition of *C. chlorophyti* and *C. siamense* responsible for postharvest anthracnose, *M. phaseolina* which causes charcoal rot, and *F. sulawesiense* which causes fruit rot in culinary melon at recommended concentrations, more precise and comprehensive evaluations, such as determining the half-maximal effective concentration (EC_50_), conducting conidial germination assays, and evaluating a broader range of fungicide classes, should be included in future studies to gain deeper insights into fungicidal efficacy. Several previous studies have documented that excessive and continuous fungicide use leads to the emergence of resistant fungal strains [91,92]. Effective fungicide resistance management requires a comprehensive approach incorporating biological control methods, systematic crop rotation, strict adherence to application guidelines, and rigorous hygiene practices for tools, fields, and storage environments [93,94]. Moreover, further studies require the molecular characterization of resistance mechanisms to confirm the underlying causes of resistance and provide valuable epidemiological data to inform resistance management strategies.

## 5. Conclusions

This study provides a comprehensive investigation into the postharvest fungal diseases affecting culinary melon in northern Thailand. In the present study, *C. chlorophyti* and *C. siamense* were isolated from anthracnose lesions, *M. phaseolina* from charcoal rot lesions, and *F. sulawesiense* from fruit rot lesions on culinary melon fruits. These fungi were identified using a combination of morphological examination and multi-gene phylogenetic approaches. Pathogenicity assays demonstrated that the four species caused similar disease symptoms both under controlled inoculation conditions and during postharvest storage. Consequently, this represents the first documented report of postharvest diseases caused by *C. chlorophyti*, *C. siamense*, *F. sulawesiense*, and *M. phaseolina* affecting culinary melon in Thailand. To our knowledge, this is the first global report documenting *C. siamense* as the causative agent of anthracnose, *M. phaseolina* of charcoal rot, and *F. sulawesiense* of fruit rot in postharvest culinary melon fruit. Future research should explore the use of biological control agents and natural antifungal compounds to enhance disease control and reduce reliance on chemical fungicides. Research should also focus on the epidemiology of postharvest fruit rot of culinary melon in different regions of Thailand.

## Figures and Tables

**Table 1:** Target genes and information of primer pairs used in this study.

Target Gene	Primer Name	Direction	Primer Sequence
ITS	ITS5	Forward	5′-GGAAGTAAAAGTCGTAACAAGG-3′
	ITS4	Reverse	5′-TCCTCCGCTTATTGATATGC-3′
*act*	ACT512F	Forward	5′-ATG TGCAAGGCCGGTTTCGC-3′
	ACT738R	Reverse	5′-TACGAGTCCTTCTGGCCCAT-3′
*tub*	T1	Forward	5′-AACATGCGTGAGATTGTAAGT-3′
	T2	Reverse	5′-TAGTGACCCTTGGCCCAGTTG-3′
	T22	Reverse	5′-TCTGGATGTTGTTGGGAATCC-3′
*cal*	CL1C	Forward	5′-GAATTCAAGGAGGCCTTCTC-3′
	CL2C	Reverse	5′-CTTCTGCATCATGAGCTGGAC-3′
	CAL-228F	Forward	5′-GAGTTCAAGGAGGCCTTCTCCC-3′
	CAL-737R	Reverse	5′-CATCTTTCTGGCCATCATGG-3′
*gapdh*	GDF1	Forward	5′-GCCGTCAACGACCCCTTCATTGA-3′
	GPDHR2	Reverse	5′-CTCRGMRGCRGCCTTGATGG-3′
*rpb2*	RPB2-5F2	Forward	5′-GGGGWGAYCAGAAGAAGGC-3′
	RPB2-7cR	Reverse	5′-CCCATRGCTTGYTTRCCCAT-3′
*tef1-α*	EF1	Forward	5′-ATGGGTAAGGARGACAAGAC-3′
	EF2	Reverse	5′-GGARGTACCAGTSATCATG-3′
	EF1-728F	Forward	5′-CATCGAGAAGTTCGAGAAGG-3′
	EF1-986R	Reverse	5′-TACTTGAAGGAACCCTTACC-3′

**Table 2:** GenBank accession numbers and BLAST results revealed the highest sequence similarity between the fungal strains in this study and ex-type strains available in the GenBank database.

Fungal Strain	Gene	GenBank Accession Number	The Closely Related Ex-Type Strain/ Similarity Value (%)
SDBR-CMU540	ITS	PV789656	*Colletotrichum siamense* ICMP 18578/99.82
	*gapdh*	PV796144	*Colletotrichum siamense* ICMP 18578/99.55
	*cal*	PV796147	*Colletotrichum siamense* ICMP 18578/99.56
	*act*	PV796150	*Colletotrichum siamense* ICMP 18578/100.00
	*tub*	PV796152	*Colletotrichum siamense* ICMP 18578/99.57
SDBR-CMU541	ITS	PV789657	*Colletotrichum siamense* ICMP 18578/99.82
	*gapdh*	PV796145	*Colletotrichum siamense* ICMP 18578/99.55
	*cal*	PV796148	*Colletotrichum siamense* ICMP 18578/99.56
	*act*	PV796151	*Colletotrichum siamense* ICMP 18578/100.00
	*tub*	PV796153	*Colletotrichum siamense* ICMP 18578/99.54
SDBR-CMU542	ITS	PV789658	*Colletotrichum chlorophyti* IMI 103806/99.81
	*gapdh*	PV796146	*Colletotrichum chlorophyti* IMI 103806/100
	*act*	PV796149	*Colletotrichum chlorophyti* IMI 103806/99.01
	*tub*	PV796154	*Colletotrichum chlorophyti* IMI 103806/99.60
SDBR-CMU543	ITS	PV789659	*Macrophomina phaseolina* CBS 205.47/100.00
	*act*	PV796155	*Macrophomina phaseolina* CBS 205.47/99.62
	*cal*	PV796158	*Macrophomina phaseolina* CBS 205.47/99.64
	*tef1-α*	PV796161	*Macrophomina phaseolina* CBS 205.47/98.57
SDBR-CMU544	ITS	PV789660	*Macrophomina phaseolina* CBS 205.47/100.00
	*act*	PV796156	*Macrophomina phaseolina* CBS 205.47/99.62
	*cal*	PV796159	*Macrophomina phaseolina* CBS 205.47/99.64
	*tef1-α*	PV796162	*Macrophomina phaseolina* CBS 205.47/98.58
SDBR-CMU545	ITS	PV789661	*Macrophomina phaseolina* CBS 205.47/100.00
	*act*	PV796157	*Macrophomina phaseolina* CBS 205.47/99.62
	*cal*	PV796160	*Macrophomina phaseolina* CBS 205.47/99.64
	*tef1-α*	PV796163	*Macrophomina phaseolina* CBS 205.47/98.58
SDBR-CMU546	*tef1-α*	PV796164	*Fusarium mianyangense* LC15879/99.10
	*cal*	PV796166	*Fusarium sulawesiense* InaCC F940/100.00
	*rpb2*	PV796168	*Fusarium sulawesiense* InaCC F940/100.00
SDBR-CMU547	*tef1-α*	PV796165	*Fusarium mianyangense* LC15879/99.10
	*cal*	PV796167	*Fusarium sulawesiense* InaCC F940/100.00
	*rpb2*	PV796169	*Fusarium sulawesiense* InaCC F940/100.00

## Data Availability

The DNA sequence data obtained from this study have been deposited in GenBank under accession numbers; ITS (PV789656 to PV789661), *gadph* (PV796144 to PV796146), *cal* (PV796147 to PV796149, PV796158 to PV796160, PV796166 and PV796167), *act* (PV796150, PV796151, PV796155 to PV796157), *tub* (PV796152 to PV796154), *tef1-α* (PV796161 to PV796165), and *rpb2* (PV796168 and PV796169).
